# Investigating the Impact of Chlorogenic Acid Content and Cellulose Nanoparticles on Sunflower Protein-Based Emulsions and Films

**DOI:** 10.3390/foods14050824

**Published:** 2025-02-27

**Authors:** Andresa Gomes, Lais Brito Cangussu, Rosiane Lopes Cunha, Leandro Soares de Oliveira, Adriana Silva Franca, Ana Letícia Rodrigues Costa

**Affiliations:** 1 Department of Food Engineering, School of Animal Science and Food Engineering, University of São Paulo (USP), Av. Duque de Caxias Norte, 225, Pirassununga 13635-900, SP, Brazil; 2 Food Research Center (FoRC), University of São Paulo, Rua do Lago, 250, Semi-Industrial Building, Block C, São Paulo 05508-080, SP, Brazil; 3 Instituto Federal do Mato Grosso do Sul (IFMS), Rua Salime Tanure, S/N, Coxim 79400-000, MS, Brazil; lais.cangussu@ifms.edu.br; 4 Department of Food Engineering and Technology, School of Food Engineering, University of Campinas (UNICAMP), Rua Monteiro Lobato, 80, Campinas 13083-862, SP, Brazil; rosiane@unicamp.br; 5 Programa de Pós-Graduação em Ciência de Alimentos (PPGCA), Universidade Federal de Minas Gerais, Av. Antônio Carlos, 6627, Belo Horizonte 31270-901, MG, Brazil; leandro@demec.ufmg.br (L.S.d.O.); adriana@demec.ufmg.br (A.S.F.); 6 Departamento de Engenharia Mecânica (DEMEC), Universidade Federal de Minas Gerais (UFMG), Av. Antônio Carlos, 6627, Belo Horizonte 31270-901, MG, Brazil; 7 Institute of Exact and Technological Sciences, Campus Florestal, Federal University of Vicosa (UFV), Rodovia LMG 818, km 6, Florestal 35690-000, MG, Brazil; ana.leticia@ufv.br

**Keywords:** biodegradable film, antioxidant film, plant-based protein

## Abstract

This study explores how varying chlorogenic acid levels (low—yellowish, Y; high—greenish, G) in sunflower proteins (SFs) affect the properties of eugenol-loaded oil-in-water emulsions and the resulting films, while examining the interaction of cellulose nanoparticles (from commercial (CNC) and banana peel sources (CNF)) with the film-forming matrix. This research fills gaps in literature by demonstrating how interactions among proteins, lipids, phenolic compounds, and cellulose nanoparticles influence film properties. The high chlorogenic acid content in SF reduced electrostatic repulsion between protein molecules, causing aggregation, oil droplet flocculation, and increased emulsion viscosity. The mechanical properties of emulsion-based films were significantly lower than those made with SF dispersions. Films made from low chlorogenic acid (yellowish SF) emulsions showed lower tensile strength and Young’s modulus but higher elongation at break compared to those made from high chlorogenic acid (greenish SF) emulsions. Water vapor permeability (WVP) decreased in films containing oil phases, but adding cellulose nanoparticles increased WVP. Despite this, the cellulose nanoparticles could not fully overcome the negative effects of lipid–protein interactions on mechanical properties and WVP. However, films containing eugenol exhibited significant antioxidant activity. The findings provide insights into developing sustainable, active packaging with antioxidant functionality and reduced environmental impact, opening new avenues for applications in food and other sectors requiring eco-friendly materials.

## 1. Introduction

Proteins derived from defatted meals have recently been investigated within the oil industry for their potential use as raw materials in producing edible and biodegradable films [1,2,3,4]. Among these agro-industrial proteins, those from the sunflower have gained attention due to their cost-effectiveness compared to other sources. Sunflower proteins (SFs) are derived from sunflower meal, a by-product of extracting oil from sunflower seeds, often intended for animal feed [5,6]. Sunflower meal has high protein content, ranging from 30% to 50% by weight, making sunflower protein an economically viable and sustainable alternative to other animal proteins [7,8]. However, protein products from sunflower meal contain a residual content of natural phenolic compounds, mainly chlorogenic acid, due to the strong interaction and formation of complexes between protein and these phenolic compounds [9,10]. Chlorogenic acid exhibits a variety of biological properties, such as anticarcinogenic, anti-allergenic, anti-obesity, anti-inflammatory, and antithrombotic effects, as well as hypoglycemic and hypolipidemic activities [11,12,13,14]. Furthermore, it is an excellent antioxidant and antimicrobial agent, making it a compelling compound for food applications [15,16,17,18]. However, high levels of phenolic compounds give sunflower protein a dark green color, which restricts its use in food applications [19,20]. In addition to causing undesirable color, the natural interaction of chlorogenic acid with proteins can alter their techno-functional properties, including oil and water binding capacity, emulsifying capacity, and rheological properties, which are crucial for their use as an emulsifier and film-forming matrix [8,20,21,22]. De-phenolized sunflower proteins demonstrate superior solubility, emulsifying activity, emulsion stability, dispersibility, and surface hydrophobicity compared to sunflower proteins with higher phenolic compound content [20,23,24]. However, the phenolic compound content appears not to significantly affect the key properties of films derived from sunflower protein dispersions, including thickness, density, moisture content, water vapor permeability, mechanical properties (tensile strength, elongation at break, and Young’s modulus), glass transition temperature, and phenolic release [23,24].

Currently, sunflower proteins are commercially available in two compositions: one with high phenolic content (greenish, G) and another with low phenolic content (yellowish, Y). In general, these SF products have a high protein content of 80%, high digestibility (95%), neutral flavor, and a low allergenic potential. The two principal fractions of sunflower proteins are 2S albumins (10–30%) and 11S globulins (70–90%) [25]. Despite their potential, little is known about the simultaneous effect of chlorogenic acid content on both emulsification and film-forming capabilities. Investigating how interactions between proteins, phenolics, and other components of the emulsified film-forming matrix (such as lipids and reinforcements) influence film properties could offer valuable insights into the development of plant protein-based packaging solutions.

Edible active films based on proteins have recently gained increased attention because they are an alternative to synthetic packaging materials due to their biodegradable, renewable, and environmentally friendly characteristics [26,27]. However, protein-based films have high water vapor permeability and unsatisfactory mechanical properties due to the hydrophilic nature of these biopolymers [28,29,30]. One of the strategies to overcome these challenges is the addition of lipids and nanoparticles in the film’s formulation [31,32] The development of oil-in-water (O/W) emulsions employing these materials can allow the development of a continuous polymeric network with improved properties. The selection of homogenization processes and ingredients can help ensure emulsion stability during the film drying process by reducing oil migration to the surface and forming a film with homogeneous structure. Our recent research revealed that sunflower proteins can lower the interfacial tension between water and oil phases and enhance the stability of emulsion droplets, making them more resistant to destabilization [33]. Films based on O/W emulsions are also interesting for their ability to carry hydrophobic active compounds, including antimicrobial and antioxidant agents that give the films their bioactive characteristics [2,32]. Eugenol, a colorless to yellowish liquid, is the principal phenolic component found in clove essential oil [34]. It has a density of 1.067 g/mL at 25 °C [35] and has been thoroughly investigated for its good antibacterial, antifungal, and antioxidant properties [36,37,38]. Nevertheless, its application is limited because it presents low water solubility, high volatility, and susceptibility to thermal degradation under environmental conditions during processing, utilization, and storage [39]. Therefore, several eugenol encapsulation techniques, including emulsification, have been employed to overcome these limitations [38,40].

Cellulose nanocrystals (CNCs) and nanofibers (CNFs) have been applied as additives to raise the mechanical and barrier mechanical properties of protein-based films [41,42,43,44]. Furthermore, these materials contribute to the kinetic stability of O/W emulsions by increasing the viscosity of the continuous phase and providing steric hindrance [45,46]. The effectiveness of cellulose nanoparticles as emulsifiers and reinforcing agents in films is mainly determined by their aspect ratio, size, and shape [41,42,47]. Cellulose fibers feature both amorphous and crystalline domains with a high aspect ratio, while crystalline particles are obtained by removing the amorphous regions, exhibiting a lower aspect ratio [48]. Our study used the cellulose nanofibers from tree cellulose nanocrystals (CelluForce NCC™, Canada) and banana peels to produce films’ precursor emulsions, aiming to study the effect of adding cellulose nanoparticles to the properties of SF-based films. Banana processing generates a substantial amount of cellulose-rich waste [49,50], which can be used to obtain cellulose nanofibers. Otherwise, commercial cellulose nanocrystals are devoid of amorphous domains influencing the films precursor emulsions.

Somes studies have investigated the creation of films using sunflower proteins, examining their antioxidant and antimicrobial properties in relation to varying concentrations of natural phenolic compounds, predominantly chlorogenic acids [23,24]. Other studies evaluated only the effect of de-phenolization on their physicochemical, structural, and functional properties, but no application was performed [9,20,21,22]. Still, to our knowledge, there are no published studies evaluating the effects of the phenolic compound content of the sunflower protein on the eugenol-loaded O/W emulsion characteristics and the film properties produced from these emulsions, as well as the interactions of different cellulose nanoparticles with the protein matrix and with sunflower protein-stabilized lipid droplets. Phenolic compounds can act as co-surfactants at the protein interfacial layer, altering its properties and favoring the decrease in tension between the aqueous and oily phases, which may influence the stability of O/W emulsions [51]. As mentioned, cellulose nanoparticles can also improve the emulsion stability and mechanical properties of films; furthermore, the presence of a bioactive compound-loaded lipid phase can enhance the water barrier and functionality of films. In view of these factors, our study utilized sunflower proteins with high and low chlorogenic acid content to create eugenol-loaded O/W emulsions. We assessed how the phenolic content affects the properties of these emulsions. Additionally, we developed antioxidant films using these eugenol-loaded O/W emulsions stabilized by sunflower proteins (greenish and yellowish) and cellulose nanoparticles (fiber and crystal) to determine how different film-forming emulsion compositions influence the mechanical, structural, functional, and barrier properties of the films. We hypothesize that the covalent bonding of phenolic compounds with sunflower proteins will modify the protein’s functional properties and its interactions with other components of the film-forming matrix, thus influencing the properties of both the emulsion and the film. Additionally, we hypothesize that the presence of oil droplets containing eugenol will reduce water vapor permeability and enhance the antioxidant properties of the biopolymer films, while cellulose nanoparticles will reinforce the film structure, improving the mechanical performance of the packaging.

## 2. Materials and Methods

### 2.1. Materials

The compounds and chemicals used for producing emulsions and for characterizing sunflower proteins, emulsions, and films included the following: SF concentrates with low chlorogenic acid content (yellowish: Y) and high chlorogenic acid content (greenish: G) (80% crude protein, SUNPROTEIN “Bio Technologies” LLC., Moscow, Russia); cellulose nanocrystals with a length of 91.7 ± 25.3 nm and a mean diameter of 4.3 ± 0.4 nm (CelluForce NCC™, Montreal, Canada); eugenol (C_10_H_12_O_2_; 4-allyl-2-methoxyphenol); β-mercaptoethanol; and Coomassie Brilliant Blue (Sigma Aldrich Co., St. Louis, MO, USA). The manufacturer provided the dry basis proximate composition of the SF concentrates, which consisted of 1.2% carbohydrates, 80.5% proteins, <0.5% lipids, 0.6% fibers, and 5.5% ashes. Banana peel bran was used as raw material to yield the cellulose nanofibers, which was prepared according to techniques delineated by Tibolla et al. [50]. Cellulose nanofibers (with a mean diameter and a length of 3.4 ± 1.3 nm and 1594.0 ± 191 nm, respectively) were obtained through enzymatic hydrolysis using xylanase enzyme (Novozymes, Araucária, PR, Brazil), following the procedures outlined by Tibolla et al. [52]. Sunflower oil was purchased locally (Bunge Alimentos S.A., Gaspar, SC, Brazil) and ultrapure water was produced using a Millipore Milli-Q system. All other chemicals used were of analytical grade.

### 2.2. Characterization of YSF and GSF

#### 2.2.1. Quantification of Chlorogenic Acid

The extraction procedures were established with modifications to the optimized conditions by Belguidoum et al. [53]. Briefly, 0.15 g of each sunflower protein sample powder, including both yellowish (YSF) and greenish (GSF) varieties, was subjected to an initial extraction using 4 mL of a 50% ethanol/water solution. Then, a second extraction was performed with 5 mL of a 75% ethanol/water solution. Finally, a third extraction was carried out with 2 mL of 100% ethanol for 10 min in an ultrasonic bath. Following each extraction, the samples were centrifuged at 3500 rpm for 10 min (Thermo IEC, Centra GP8R, Needham Heights, MA, USA), and the supernatants were pooled. The combined extract volume was adjusted to 10 mL with 100% ethanol. Prior to chromatographic analysis, the extracts were filtered through 0.2 μm filters.

Chlorogenic acid in the SF extracts was identified and quantified using a high-performance liquid chromatography (HPLC) system (Prominence Model, Shimadzu, Kyoto, Japan) equipped with a UV detector (203–325 nm). The analysis was conducted on a Shimadzu C18 column (5 μm, 4.6 × 150 mm) at 50 °C, following the methodology outlined by Cangussu et al. [54]. Two mobile phases were used: phase A (water/acetonitrile/phosphoric acid—92.6:7:0.4) and phase B (acetonitrile with 0.4% phosphoric acid). The flow rate used was 1.2 mL/min. Gradient elution was performed as follows: 0–8 min, 1–3% linear B; 8–12 min, 3–8% linear B; 12–15 min, 8–10% linear B; 15–20 min, 10–15% linear B; 20–25 min, 15–40% linear B; 25–30 min, 40–80% linear B; 30–35 min, 80–95% linear B; 35–35.1 min, 95–1% linear B; and 35.1–42 min, 1% isocratic B. To confirm the presence of chlorogenic acid, we compared the retention times and UV spectra of the YSF and GSF extracts with those of a chlorogenic acid standard (Sigma-Aldrich Co., St. Louis, MO, USA; min. 95%). Quantification of chlorogenic acid in the extracts was carried out using standard calibration curves.

#### 2.2.2. Solubility

The solubility profile of YSF and GSF concentrates was assessed using a revised version of the procedure outlined by Malik and Saini [20]. A 2% (*w*/*w*) protein solution was prepared and adjusted to pH levels of 3, 5, 7, 9, and 11 using 0.5 N NaOH and 0.5 N HCl. The mixtures were agitated with a magnetic stirrer for 1 h at room temperature. Following stirring, the dispersions were centrifuged at 3000 rpm for 20 min (Thermo IEC, Centra GP8R, Needham Heights, MA, USA). The supernatant was carefully separated, and its protein content was analyzed using the Kjeldahl method. Protein solubility was subsequently calculated using Equation (1).

(1) $$S\;(\%)=\frac{Protein\;content\;in\;the\;supernatant\;}{Total\;protein\;content}$$

#### 2.2.3. Zeta Potential and Particle Size of Soluble Portion of SF

Zeta potential was measured at 25 °C using the electrophoretic light scattering (ELS) technique, while particle size and polydispersity index (PDI) were obtained using dynamic light scattering (DLS). These measurements were performed with a Zetasizer Nano Series Nano ZS (Malvern Instruments Ltd., Worcestershire, UK). Samples were diluted in MilliQ water (0.001 *v*/*v*) and stabilized for 120 s before collecting data on zeta potential and particle size through three continuous readings.

#### 2.2.4. Particle Size of Insoluble Portion of SF

The particle size of the insoluble fraction of SF was assessed using static light scattering (SLS) with a Mastersizer 2000 (Malvern Instruments, Ltd., Worcestershire, UK). The samples were dispersed in water with a rotational speed of 2300 rpm, applying refractive indices of 1.33 for water and 1.48 for the protein. The average particle diameter of the insoluble fraction was reported as the volume–surface mean diameter ($D_{43}$), determined using Equation (2), and the span was obtained using Equation (3):

(2) $$D_{43}=\sum \frac{n_{i\;}D_{i}^{4}}{n_{i\;}D_{i}^{3}}$$

(3) $$Span=\frac{d_{90}-d_{10}}{d_{50}}$$

where $n_{i}$ represents the number of particles with diameter $D_{i}$ and $d_{10},d_{50},\;and\;d_{90}\;$ denote the diameters corresponding to 10%, 50%, and 90% of the cumulative volume, respectively.

#### 2.2.5. Sodium Dodecyl Sulfate Polyacrylamide Gel Electrophoresis (SDS-PAGE)

The molecular weight distribution of SF was analyzed using SDS-PAGE [55] on a 1.5 mm thick vertical slab gel (Bio-Rad Laboratories, Mini-Protean 3 Cell). YSF and GSF powders were dissolved in a 1% (*w*/*v*) aqueous SDS solution to achieve protein concentrations of 6 mg/mL and 8 mg/mL, respectively. Sample buffer (50 mM Tris/HCl, pH 6.8, 2% SDS, 0.05% bromophenol blue, 20% glycerol, and 2% β-mercaptoethanol or water) was mixed with each sample, which was subsequently heated in a water bath at 95 °C for 4 min. Then, 10 μL of each sample was loaded onto the polyacrylamide gel, which consisted of 15% acrylamide for the separating gel and 5% acrylamide for the stacking gel. Electrophoresis was performed at a constant voltage of 100 V. Following this, the gels were stained with 0.025% (*w*/*v*) Coomassie Brilliant Blue in a solution of ethanol, acetic acid, and water (45:10:45 *v*/*v*) for 12 h, succeeded by de-staining with repeated washes in a solution of ethanol, acetic acid, and water (10:5:85 *v*/*v*). A pre-stained protein standard (BenchMark™ Protein Ladder, Thermo Scientific, Waltham, MA, USA), with molecular weights ranging from 6 to 180 kDa, was used for reference.

#### 2.2.6. Differential Scanning Calorimetry (DSC)

Thermograms for the YSF and GSF were obtained using differential scanning calorimetry (DSC) with a TA 2010 differential scanning calorimeter (TA Instruments, New Castle, DE, USA), operated through a TA5000 system and equipped with a cryogenic quench cooling accessory. The protein powders were dissolved in distilled water at a concentration of 20% (*w*/*w*) for 30 min before analysis, with the pH adjusted to 11 using 1 M NaOH. The samples were placed in hermetically sealed aluminum pans and heated at a rate of 10 °C/min over a temperature range from 0 to 110 °C, using an empty pan as a reference for comparison [56]. The melting temperature and enthalpy were determined directly from the DSC curves using Universal Analysis V1.7F software (TA Instruments).

### 2.3. Emulsion Production

The aqueous phase of emulsions was comprised of dispersions containing YSF or GSF (5% *w*/*w*; real protein content 4% *w*/*w*), glycerol (1.5% *w*/*w*), and cellulose nanofibers (CNFs; 0.05% *w*/*w*) or cellulose nanocrystals (CNCs; 0.05% *w*/*w*), while the oil phase was composed of sunflower oil (3.5% *w*/*w*) and eugenol (1.5 % *w*/*w*).

Sunflower proteins (YSF and GSF) were dissolved in water using a magnetic stirrer at 25 ± 2 °C for 5 min. The pH of the dispersions was adjusted to pH 11 by adding 2 M NaOH while stirring continuously for 30 min. During this period, the pH was tracked and adjusted as needed every 5 min. After solubilizing sunflower proteins, glycerol and CNC or CNF particles were added to the solution and stirred with a magnetic stirrer until fully dissolved, which took approximately 2 h.

**Figure 1:**
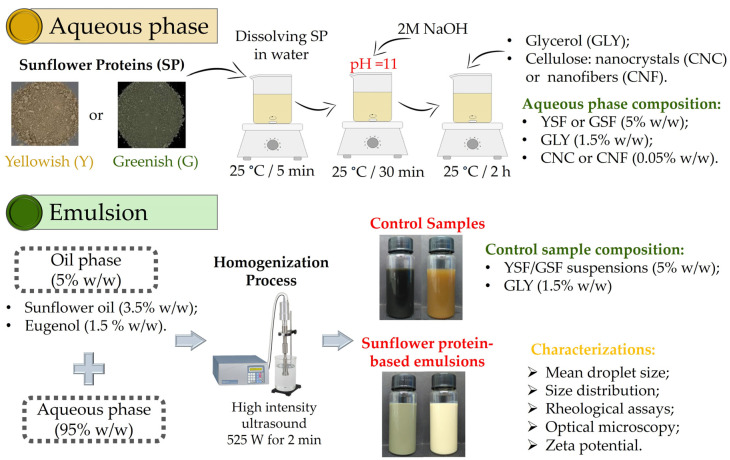
Schematic diagram of the preparation process of sunflower protein-based emulsions.

All O/W emulsions (70 g) were prepared using a fixed weight ratio of 5% oil phase to 95% aqueous phase. The dispersions were homogenized with a QR 750 W ultrasonic probe (Ultronique, Indaiatuba, Brazil) operating at a frequency of 20 kHz with a 13 mm diameter titanium probe. The nominal power during emulsification was 525 W, and the homogenization was carried out for 2 min. The dispersions of YSF or GSF, composed of 5% *w*/*w* of protein (real protein content 4 % *w*/*w*) and 1.5% glycerol (*w*/*w*), were used as control samples, called C-YSF and C-GSF. Two replicates of each sample were prepared and characterized as detailed in Section 2.4. Figure 1 presents a schematic diagram of the emulsion preparation process, while Table 1 outlines the composition of the film-forming emulsions evaluated in this study.

### 2.4. Characterization of Emulsions

#### 2.4.1. Mean Droplet Size, Size Distribution, and Rheological Assays

The technique of laser diffraction using a Mastersizer 2000 (Malvern Instruments Ltd., Worcestershire, UK) was employed to obtain the curves of mean droplet size and size distribution of emulsions, according to Costa et al. [45]. Measurements were taken right after the preparation of the emulsions. The mean droplet size was defined as the volume–surface mean diameter ($D_{32}$), calculated using Equation (4), while the Span was determined according to Equation (3) (Section 2.2.4):

(4) $$D_{32}=\sum \frac{n_{i\;}D_{i}^{3}}{n_{i\;}D_{i}^{2}}$$

where $n_{i}$ represents the number of particles with diameter $D_{i}$.

After preparation of the emulsions, rheological assessments were made at 25 °C using a stress-controlled rheometer (AR1500ex, TA Instruments, Leatherhead, UK) with stainless steel cone–plate geometry (cone truncation 208 μm, diameter 60 mm, and angle 2°), according to Costa et al. [45]. The shear rate was altered within the range of 0.1 to 300 s^−1^, and flow curves were obtained through a three-step sequential flow process (up–down–up cycles). The data from the third flow curve were analyzed using the power law model, described by Equation (5):

(5) $$\sigma =k\times ({\dot{\gamma })}^{n}$$

where σ represents the shear stress (Pa), $\dot{\gamma }$ denotes the shear rate (s^−1^), k is the consistency index (Pa·s^n^), and n is the flow behavior index (dimensionless).

#### 2.4.2. Optical Microscopy and Zeta Potential

An optical microscope (Axio Scope.A1, Carl Zeiss, Hilden, Germany) equipped with a 100× oil immersion objective lens was employed to examine the microstructure of emulsions at 25 °C. Images were captured using AxioVision Rel. 4.8 software (Carl Zeiss, Germany).

Initially, samples were diluted in MilliQ water to a concentration of 0.001 vol %, and the pH was adjusted to 11 by adding 0.1 M NaOH. The zeta potential of samples was analyzed using a Zeta Sizer Nano Series (Malvern Instruments, Worcestershire, UK). Each sample was stabilized for 120 s in the instrument before collecting data through five continuous readings.

### 2.5. Film Production

Sixty milliliters (60 mL) of each O/W emulsion sample were poured into polypropylene trays for casting the films. The trays containing the emulsion films were placed in a convection oven at a controlled temperature of 25 °C. After 12 h, the dry films were carefully removed from the trays. Then, they were then placed in a desiccator at room temperature for 2 days, maintaining a relative humidity of 58%. Each film sample was produced in triplicate. Thereafter, all films were characterized as described in Section 2.6. The films made solely from SF dispersions (yellowish or greenish) were used as control samples and were characterized using the same techniques as those applied to the emulsion-based films.

### 2.6. Characterization of Films

#### 2.6.1. Mechanical Properties, Thickness, and Water Vapor Permeability (WVP)

The mechanical properties of the sunflower protein-based films were evaluated following ASTM standard D882-12 (ASTM, 2012) [57]. Ten readings were averaged for each sample [58]. The elongation at break and tensile strength (force per initial cross-sectional area) of the films were measured using a TA.TX Plus Texture Analyzer (Stable Micro Systems, Surrey, UK). Strength versus elongation curves were analyzed with Texture Exponent 32 software, and the Young’s modulus was determined from the slope of the initial linear segment of these curves (approximately 0.5% strain).

The thickness of the films was determined by averaging measurements taken at 10 random locations on the film samples using a digital micrometer (Digimatic Micrometer Series 293 MDC-Lite, Mitutoyo Corporation, Kawasaki-shi, Japan).

The experiment used triplicate measurements of gravimetric processing to ascertain the WVP of films. The procedure followed standard method E96-00/ E96M-24a (ASTM, 2000) [59] with some modifications [58]. The WVP of the films was determined by Equation (6) and reported in g/m.s.Pa:

(6) $$WVP=\frac{w}{t}\;.\;\frac{\delta }{∆P\times A}$$

where  w/t is the slope of the weight gain (w) over time (t) in (g/s); δ represents the average sample thickness (m); A is the sample permeation area (m^2^); and ∆P (Pa) is the difference in water vapor pressure across the sample, ranging from ambient conditions with 64% to 33% relative humidity at 25 °C.

#### 2.6.2. Color and Scanning Electron Microscopy (SEM)

Film color was measured in a tristimulus colorimeter (ColorFlex, Hunter Associates Laboratory, Reston, VA, USA), in reflectance mode, with D65 standard illumination and a 10° observer angle. Color measurements were reported in terms of lightness L* (L* = 0 represents black and L* = 100 represents white) and chromaticity parameters a* (ranging from green [−] to red [+]) and b* (ranging from blue [−] to yellow [+]). The L*, a*, and b* data values were used to calculate chroma (C*), which indicates the color intensity, and the hue angle (h), which represents the color tone in degrees (0° for red, 90° for yellow, 180° for green, and 270° for blue). The chroma (C*) and hue angle (h) were determined using Equation (7) and Equation (8), respectively.

(7) $$C^{*}=\sqrt{{\left(a^{*}\right)}^{2}+{\left(b^{*}\right)}^{2}}$$

(8) $$h={tan}^{-1}\left(\frac{b^{*}}{a^{*}}\right)$$

The film’s surfaces were examined using scanning electron microscopy (SEM). First, the film samples were sectioned and fixed onto aluminum stubs using double-sided carbon tape. Subsequently, the samples were coated with a thin gold layer to enhance conductivity and were examined with a scanning electron microscope (FEI Quanta 200 FEG, Field Electron and Ion Company, FEI, Waltham, MA, USA) at an acceleration voltage of 5 kV.

#### 2.6.3. Antioxidant Activity and Total Phenolic Content

Film samples (2 cm in diameter, 0.1 g) were immersed in 250 mL flasks with 25 mL of 50% ethanol (*v*/*v*). The flasks were sealed and agitated at 100 rpm at room temperature for 24 h. After the films were removed, the resulting extracts were analyzed to determine their antioxidant activity and total phenolic content.

The total phenolic content (TP) was quantified using a modified Folin–Ciocalteu colorimetric assay, with results reported as grams of gallic acid equivalents (GAEs) per 100 g of film [60].

For the ferric reducing antioxidant power (FRAP) assay, a reagent was prepared by combining 100 mL of acetate buffer (0.3 M), 10 mL of a TPTZ (10 mM) solution in HCl (40 mM), and 10 mL of aqueous ferric chloride (20 mM). To this reagent, 2.7 mL was mixed with 90 μL of each extract and 270 μL of distilled water. The mixture was incubated at 37 °C for 30 min, and the absorbance was measured at 595 nm. A calibration curve was constructed using ferrous sulfate (500–2000 μM), and the results were reported in mmol Fe_2_SO_4_ per gram of film.

The ABTS•+ assay was performed based on the method by Rufino et al. [61], with minor adjustments. ABTS•+ radicals were produced by oxidizing a 7 mM ABTS (2,2′-azinobis [3-ethylbenzothiazoline-6-sulfonic acid] diammonium salt) solution with 145 mM potassium persulfate. This mixture was incubated in the dark at room temperature for 12–16 h. Prior to use, the ABTS•+ solution was diluted with ethanol to achieve an absorbance of 0.70 ± 0.02 at 734 nm. Subsequently, 3 mL of the ABTS•+ solution was mixed with 30 μL of each diluted extract. After a 6 min reaction, the absorbance was measured at 734 nm against a blank consisting of 30 μL of the diluted extract and 3 mL of ethanol. The results were reported as mmol Trolox (6-hydroxy-2,5,7,8-tetramethylchroman-2-carboxylic acid) equivalents (TEs) per gram of film.

### 2.7. Statistical Analysis

Each experiment was conducted in duplicate, with a minimum of three measurements per sample. Statistical evaluation was carried out using Minitab 16^®^ software, employing analysis of variance (ANOVA) and Tukey’s test to determine significant differences (*p* < 0.05) among the treatments.

## 3. Results and Discussion

### 3.1. YSF and GSF Properties

**Figure 2:**
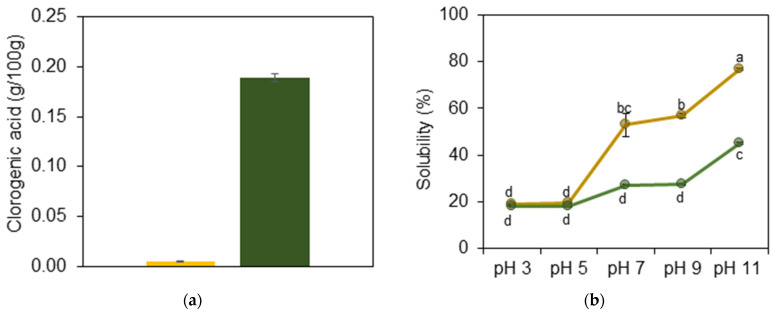

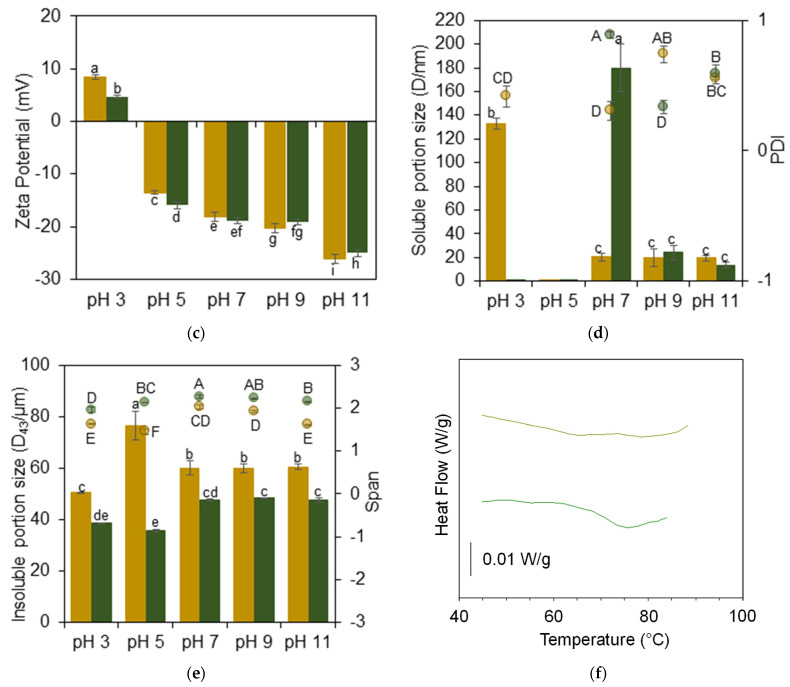
Characterization of sunflower proteins (SPs) with low (yellowish, Y) and high (greenish, G) chlorogenic acid content: (**a**) chlorogenic acid content; (**b**) solubility; (**c**) zeta potential; (**d**) particle size (bars) and polydispersity index (PDI, dots) of the soluble portion of sunflower proteins; (**e**) mean particle size (D_43_, bars) and span (dots) of the insoluble portion of sunflower proteins; (**f**) DSC thermograms of yellowish sunflower protein (∎) and greenish sunflower protein (∎). Means that do not share a letter are significantly different.

Sunflower meal contains phenolic compounds ranging from 1 to 4% by weight, with chlorogenic acid being predominant [6,62]. In the traditional alkaline extraction method used to obtain oil-based proteins, chlorogenic acid molecules undergo oxidation to form quinones. These quinones then interact with protein functional groups, causing them to co-precipitate during the isoelectric precipitation phase [19,63]. The sunflower protein used in our study, characterized by a low chlorogenic acid content (yellowish), was commercially produced by eliminating phenolic compounds through a clarification process (“Bio Technologies” LLC., Russia). Thus, as anticipated, the content of chlorogenic acid in the commercial GSF was higher than in the YSF, as shown in Figure 2a.

In this study, we selected sunflower protein with varying levels of chlorogenic acid. We will examine how the content of phenolic compounds influences the properties of emulsions and films. Yellowish films are ideal for applications where color matters, such as packaging for minimally processed vegetables, whereas greenish films are more suitable for applications where color is less of a concern, such as agricultural plastics [23].

As presented in Figure 2b and reported by previous studies, YSF and GSF show reduced solubility close to their isoelectric point (pI = 5) and increased solubility at alkaline pH levels, with maximum solubility observed at pH 11 [20,64]. YSF demonstrated a solubility value of 77%, which was significantly higher than the 45% solubility of GSF. The reduced solubility of GSF was attributed to the strong interactions between chlorogenic acids and protein molecules that resulted in the formation of protein–phenolic complexes [9,10]. Low solubility in water hinders the displacement of protein molecules to the oil–water interface, thereby reducing their emulsifying capacity and, consequently, effective emulsion stabilization [20,65].

Raising the pH led to a notable increase in the solubility of the proteins, which in turn caused a significant change in the zeta potential. For YSF, the zeta potential shifted from +8.41 mV to −26.15 mV, while for GSF it changed from +4.64 mV to −25.03 mV. This increase in zeta potential induced electrostatic repulsion between the protein molecules, reducing aggregation and decreasing the soluble portion sizes from 132.68 nm to 19.55 nm for YSF and from 179.84 nm to 13.77 nm for GSF (Figure 2c–e). Based on these results, pH 11 was selected for our experiments.

SDS-PAGE analysis of YSF (C) and GSF (G) under both reducing (+) and non-reducing (-) conditions revealed bands with molecular masses between 19–37 kDa and 37–49 kDa, corresponding to the basic (β) and acidic (α) polypeptides, respectively (Figure S1—Supplementary Materials). Additionally, both YSF and GSF samples showed a high molecular weight aggregate (above 94 kDa) that did not pass through the resolving gel. The 2S albumins, which usually range from 14 to 18 kDa and are known for their high water solubility, were likely removed during the aqueous extraction of phenolic compounds [9]. Notably, only the YSF from the non-reducing condition showed 11S globulins with αβ subunits around 64 kDa.

The DSC thermograms for YSF and GYS at pH 11 displayed a single endothermic peak, indicating the denaturation temperature, as depicted in Figure 2f. YSF showed higher denaturation temperature (81.18 ± 1.29 °C) and lower enthalpy value (1.46 × 10^−4^ ± 1.28 × 10^−6^ J/g) than GSF (71.98 ± 4.97 °C; 2.29 × 10^−4^ ± 6.97 × 10^−5^ J/g). The denaturation temperatures are consistent with previous studies, though the enthalpy values are significantly lower, suggesting a higher degree of denaturation and reduced crosslinking between protein molecules [20]. The degree of denaturation of YSF was higher than that of GSF, likely due to the additional de-phenolization process. Like many commercial proteins, sunflower protein has undergone some degree of denaturation during its extraction process. Additionally, the DSC thermograms were performed on protein dispersions at pH 11, which may have also contributed to lower denaturation enthalpy values. Protein denaturation leads to increased hydrophobicity and flexibility, which are essential characteristics for effective emulsifiers [66]. The increased hydrophobicity and flexibility of de-phenolized proteins were primarily evidenced by their enhanced solubility. This improvement was directly correlated with their emulsifying performance, as demonstrated by higher zeta potential values and reduced droplet size. Detailed analysis and supporting data for these relationships are provided in the subsequent sections.

### 3.2. Emulsion Properties

**Figure 3:**
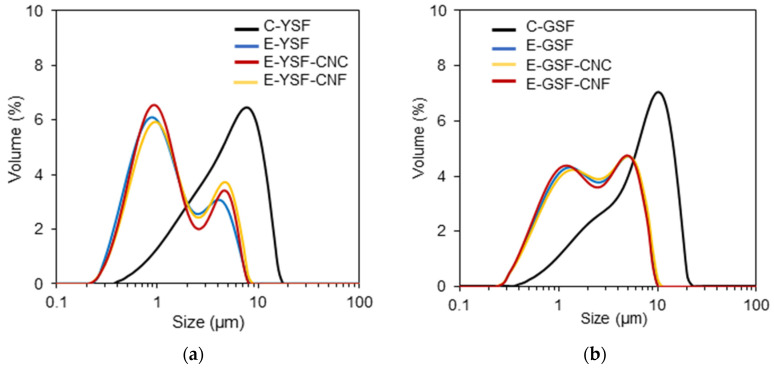
(**a**) Particle size distribution curves of samples produced with yellowish sunflower protein: dispersion (C-YSF), emulsion (E-YSF), emulsion added with cellulose nanocrystals (E-YSF-CNC), or cellulose nanofibers (E-YSF-CNF); (**b**) particle size distribution curves of samples of greenish sunflower protein: dispersion (C-GSF), emulsion (E-GSF), emulsion added with cellulose nanocrystals (E-GSF-CNC), or cellulose nanofibers (E-GSF-CNF).

Figure 3 presents the particle size distribution curves for C-YSF and C-GSF dispersions, as well as the droplet size distributions for emulsions produced only with protein dispersions (E-YSF and E-GSF), and emulsions prepared with both protein dispersions and cellulose nanocrystals (E-YSF-CNC and E-GSF-CNC) or cellulose nanofibers (E-YSF-CNF and E-GSF-CNF). The C-YSF and C-GSF dispersions exhibited a wide particle size distribution, in which the D_32_ values were, respectively, 3.52 and 2.95 μm. Sunflower protein isolates had a larger particle size (45 μm) compared to sunflower protein concentrates, indicating that the isolate was more prone to molecular interactions and the formation of larger aggregates with phenolic compounds [23].

**Figure 4:**
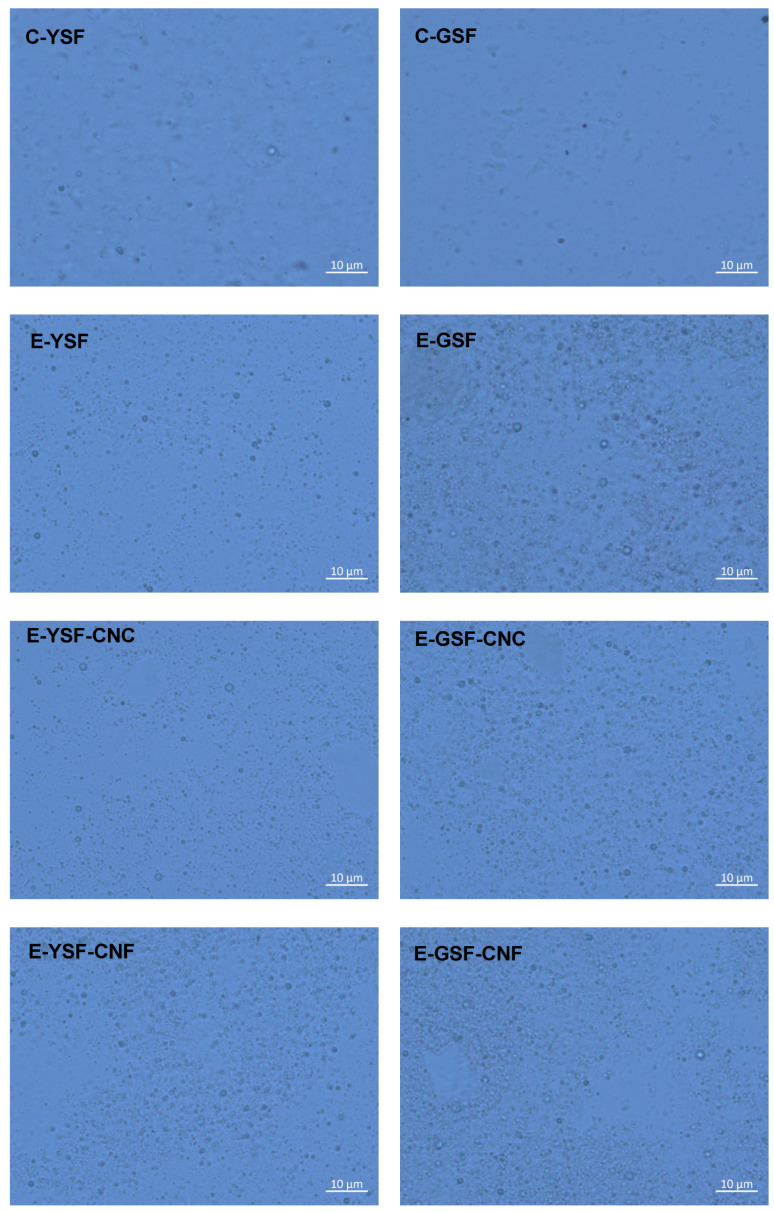
Optical microscopy of yellowish (C-YSF) and greenish (C-GSF) sunflower protein dispersions, emulsions prepared only with the yellowish (E-YSF) and greenish (E-GSF) sunflower protein dispersions, and sunflower protein-based emulsions added with cellulose nanocrystals (E-YSF-CNC or E-GSF-CNC) or cellulose nanofibers (E-YSF-CNF or E-GSF-CNF).

All emulsions displayed bimodal droplet size distributions with smaller particles compared to the corresponding C-YSF and C-GSF dispersions, suggesting that the SF molecules may unfold and adsorb at the O/W interface. E-YSF had a more pronounced peak at smaller sizes (1 μm) and a lower volume peak at bigger sizes (5 μm), whereas E-GSF showed these two peaks with nearly equal volume. The removal of phenolics enhances the solubility and emulsifying capacity of sunflower protein, which can contribute to the production of emulsified systems with smaller droplet diameters [20,67]. Although oil droplets stabilized by YSF were smaller (0.9–0.98 µm) than those stabilized by GSF (1.34–1.38 µm), the E-YSF droplets were more polydisperse (span ≈ 3.5) (Table 2). Micrographs of all emulsions primarily revealed small oil droplets (diameter around 1 μm) (Figure 4). Consequently, the presence of larger size peaks is probably attributable to the formation of protein clusters within the continuous phase, which promotes aggregation among oil droplets. Research indicates that phenolic compounds can oxidize to form o-quinones, which are highly reactive and can covalently bond with the amino (-NH2) groups of proteins at high pH. This interaction reduces the zeta potential and hydrophobicity of sunflower protein, thereby limiting its unfolding at the water–oil interface and leading to increased protein aggregation in the continuous phase [9,20,68,69].

The original YSF and GSF dispersions and their emulsions presented shear thinning behavior, which was accurately described by the power-law model (Table 2). Emulsions made with GSF demonstrated more pronounced increases in viscosity and shear thinning (ƞ at 10 s^−1^ = 135.2–296.5 Pa.s; n = 0.54–1.59) compared to those made with YSF (ƞ at 10 s^−1^ = 29.5–86.8 Pa.s; n = 0.67–0.79). This higher viscosity and increased resistance to flow in GSF emulsions could be attributed to more significant aggregation and flocculation of droplets (D_32_ values in Table 2). As already stated, the reduced electrostatic repulsion (lower zeta potential values) likely enhances attractive interactions between protein molecules, promoting the flocculation of oil droplets [70].

Both YSF and GSF dispersions exhibited negative zeta potential values (around −33 mV) due to the pH of the solutions (pH = 11) being above the protein’s isoelectric point (pI = 5) [25]. However, emulsions made with YSF had higher zeta potential values (35.7–37.15 mV) compared to those made with GSF (33.67–34.98 mV), leading to lower D_32_ and viscosity measurements. The incorporation of cellulose nanoparticles enhanced the negative zeta potential in all SF emulsions (Table 2). The negative charge of the CNCs is due to sulfate groups introduced onto the cellulose chains through sulfuric acid treatment. On the other hand, the negative charge on CNFs was generated by increased exposure to oxygen during the ultrasound homogenization process, which promoted partial oxidation [45,71].

### 3.3. Film Properties

**Figure 5:**
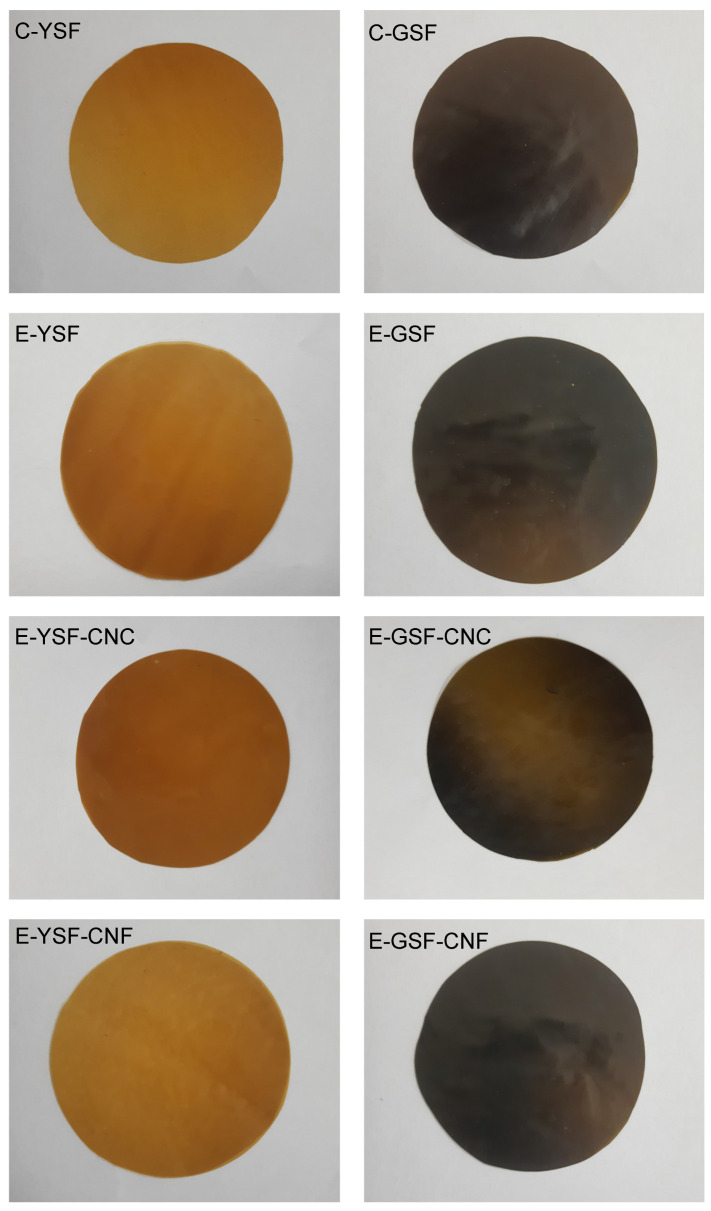
Films obtained from yellowish (C-YSF) and greenish (C-GSF) sunflower protein dispersions, emulsions prepared only with the yellowish (E-YSF) and greenish (E-GSF) sunflower protein dispersions, and sunflower protein-based emulsions added with cellulose nanocrystals (E-YSF-CNC or E-GSF-CNC) or cellulose nanofibers (E-YSF-CNF or E-GSF-CNF).

All films displayed good visual quality and uniform color (Figure 5), indicating that no significant phase separation occurred in the film-forming emulsions during the drying process (Figure S2—Supplementary Materials). As shown in Table 3, the thickness of films made from YSF or GSF dispersions was significantly lower than films made from emulsions. The incorporation of solids into the polymeric matrix, particularly with the incorporation of hydrophobic substances such as eugenol and sunflower oil, led to thicker films [29,32,72].

Color significantly influences the acceptability of food products by consumers [2]. In the face of this, the color parameters of films were carefully examined (Table 4). Overall, the lightness (L*) of all films was notably low due to the light absorption by natural phenolic compounds and eugenol (when added). Although all films tended to exhibit a yellow tone, indicated by h values close to 90° (except for C-YSF), the yellow tone was less pronounced in films produced with GSF, rich in chlorogenic acid. Films based on GSF appeared darker and duller, as evidenced by their lower C* and L* values. This darker color is due to the oxidation of chlorogenic acid to o-quinones during the protein extraction process in an alkaline environment [23]. Conversely, the control film with low chlorogenic acid content (C-YSF) had lower color saturation and a more neutral appearance (lowest h and C* values).

Films containing emulsions appeared lighter and had a more pronounced yellow tone. While the color parameters of films made with YSF were enhanced with the incorporation of oil, films made with GSF showed a decrease in these values, except for the a* parameter, which remained unchanged. The addition of sunflower oil also did not significantly alter the color of films obtained from emulsions stabilized by whey protein isolate [73]. However, the sunflower oil droplets dispersed within the film-forming matrix, composed of quinoa protein and chitosan, interacted with water molecules, altering the refractive index of the hydrocolloids, and, as a result, changed the color characteristics of the films [29]. This suggests that the addition of oil can either maintain the film color or induce alterations on color parameters, depending on the intensity of interactions between oil droplets and the film-forming matrix. Notably, cellulose nanoparticles showed negligible influence on the color of films derived from YSF and GSF. The incorporation of montmorillonite into gelatin-based films activated with ginger essential oil nanoemulsion did not affect lightness (L*) or redness (a*); however, a slight increase in yellowness (b*) was observed [74]. Similarly, the addition and increasing concentration of 2,2,6,6-tetramethylpiperidine-1-oxyl radical-oxidized cellulose nanocrystals in ginger essential oil emulsions did not alter the L* or a* values of mung bean starch composite films, but a slight decrease was noted in b* values [75].

**Figure 6:**
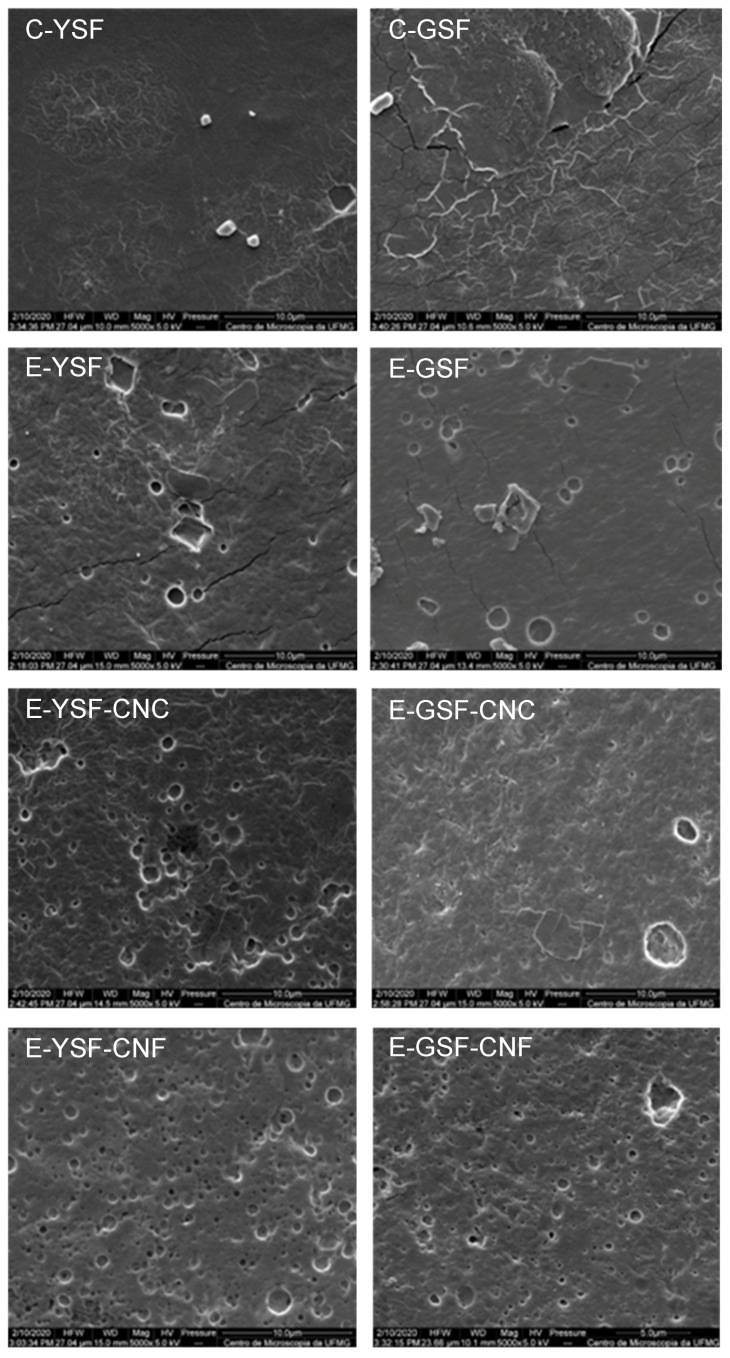
SEM of films obtained from yellowish (C-YSF) and greenish (C-GSF) sunflower protein dispersions, emulsions prepared only with the yellowish (E-YSF) and greenish (E-GSF) sunflower protein dispersions, and sunflower protein-based emulsions added with cellulose nanocrystals (E-YSF-CNC or E-GSF-CNC) or cellulose nanofibers (E-YSF-CNF or E-GSF-CNF).

The durability of films and their capacity to preserve the mechanical integrity of food are strongly associated with their mechanical properties [76]. The tensile strength and Young’s modulus of the control film based on GSF were significantly lower than that made with YSF, though its elongation at break was higher (Table 3). Some studies have demonstrated that films produced from SF naturally linked to phenolic compounds exhibited similar mechanical behavior to those made from de-phenolized SF or with added essential oils [5,23]. Therefore, the mechanical properties observed in our study were linked to the microstructural heterogeneity of the C-GSF film (Figure 6). The C-YSF film exhibited a more uniform and smoother surface, whereas the irregularities found in the C-GSF film resulted in a weaker and more flexible polymeric matrix.

Overall, the tensile strength and Young’s modulus of E-YSF and E-GSF films were significantly lower than those made from sunflower protein dispersions. The presence of oil droplets likely enhanced lipid–protein interactions while weakening protein–protein interactions [2,29,73]. This led to discontinuities in the polymeric matrix, resulting in reduced film cohesion and mechanical strength [77]. Figure 6 presents the micrographs of emulsion-based films, in which it is possible to clearly observe the presence of cracks that appear to have originated from micropores, possibly linked to the presence of oil droplets. These cracks may have propagated abruptly throughout the film, resulting in reduced strength and stiffness. The presence of oil droplets did not influence the elongation at break of the E-YSF films, but a significant reduction in this property was observed in E-GSF films. Although the disruption of protein chain interactions facilitates chain displacement during stretching, the presence of cracks (Figure 6) reduces the film’s ability to deform without breaking.

Interestingly, films based on E-YSF exhibited lower tensile strength and Young’s modulus but higher elongation at break compared to those based on E-GSF. In emulsified systems, proteins can act as emulsifiers, forming a layer around droplets with different rheological behaviors depending on the emulsifier’s characteristics and its interactions with hydrophilic and hydrophobic phases [78]. Some interfacial layers may interact minimally with the film-forming matrix without significantly affecting the rheology of the bulk system. Conversely, the interface can interact with the film-forming matrix network, enhancing the system’s viscoelasticity [79]. Interfaces composed of de-phenolized sunflower protein isolate were reported to form a viscoelastic interfacial network, while no viscoelastic behavior was observed when natural sunflower protein isolate, rich in chlorogenic acid, was used [21]. Therefore, we can suggest that the interfacial layer composed of YSF formed a more viscoelastic interfacial layer around the oil droplets. This layer was able to connect to the gel network of the film-forming matrix, facilitating the development of a more flexible polymeric structure.

The presence of cracks in emulsified films raises concerns regarding their performance in practical applications. For instance, in food packaging, such defects could compromise the barrier properties, potentially allowing moisture or oxygen penetration, which would reduce the shelf life of packaged products. Similarly, in biomedical applications, cracks could reduce the film’s ability to provide consistent mechanical support or protection, limiting its use in wound dressings or drug delivery systems. Conversely, the increased elongation at break observed in certain film formulations may make them suitable for applications where flexibility is prioritized over mechanical strength, such as wraps for fresh produce. The microstructural features observed in SEM images directly influence the mechanical properties and potential applications of the films. Tailoring film compositions to minimize defects like cracks while maintaining desired flexibility and strength is essential for optimizing their performance in specific applications, whether in food packaging, where barrier properties are crucial, or in biomedical settings requiring flexibility and biocompatibility.

Films containing cellulose nanoparticles showed no visible cracks, despite an increased number of micropores caused by oil droplets (Figure 6). The incorporation of CNC and CNF particles had no significant impact on the tensile strength or Young’s modulus of emulsified films; however, a notable decrease in elongation at break was observed, indicating reduced film flexibility. Several studies have reported that CNC and CNF particles can enhance the tensile strength and Young’s modulus of protein films through particle–protein interactions and the filling effects of cellulose nanoparticles within the polymeric matrix [41,42,43]. However, cellulose particles appear to behave differently in films containing emulsified oil droplets. Cellulose nanoparticles can act at the oil–water interface, contributing to emulsion stabilization [80,81]. The addition of cellulose nanoparticles likely formed a more structured robust interface around the oil droplets, preventing crack formation in our emulsified films. Consequently, their role in the bulk structure, as fillers and reinforcement agents, was minimized, resulting in negligible improvements in tensile strength or Young’s modulus. However, the production of more structured and homogeneous films may explain the observed decrease in elongation at break. Conversely, the addition of montmorillonite to gelatin-based films activated with ginger essential oil nanoemulsion did not affect the Young’s modulus and elongation at break but resulted in an increase in tensile strength [74]. Therefore, the negative impact of lipid–protein interactions on the film’s cohesion could not be fully offset by the interactions between cellulose nanoparticles and proteins. As a result, no significant improvement in the film’s mechanical properties was noted.

As previously mentioned, the higher phenolic content in sunflower protein results in darker and less flexible films, which may not be ideal for certain food packaging applications. These properties can limit their functionality and consumer appeal, particularly in markets where visual appearance and flexibility are key considerations. To overcome these limitations, one potential approach is optimizing the sunflower protein extraction process to reduce phenolic content while preserving protein yield. Another strategy involves incorporating natural or synthetic additives, such as bio-based reinforcements, to enhance the structural integrity and flexibility of the films. Additionally, integrating phenolic compounds with antioxidants or other bioactive substances (e.g., eugenol) could result in multifunctional packaging materials with added health benefits, making them suitable for applications as primary films. Films with higher phenolic content may still find utility in applications where visual appearance is less critical, such as bulk or industrial packaging for non-food items. Furthermore, the antioxidant properties of phenolic compounds could be advantageous for packaging products that require an extended shelf life or enhanced protection against oxidative degradation, such as oils, nuts, and dried fruits.

Consumer acceptability is a critical factor when developing new packaging materials. For food-related applications, visual appeal plays a significant role in influencing consumer perception and purchase decisions. Darker films, resulting from higher phenolic content, may be perceived as less attractive or less natural, potentially impacting on their marketability. To address this, transparent or lightly tinted films based on de-phenolized sunflower protein can be developed to align better with consumer expectations for premium or visible food products. Additionally, the inclusion of bioactive compounds or antioxidants (e.g., eugenol) that provide tangible health benefits—such as prolonging the shelf life of packaged foods—can enhance consumer perception by emphasizing the functional and sustainable aspects of the packaging. Communicating these benefits clearly through labeling and marketing can further improve acceptance, particularly among environmentally conscious and health-oriented consumers.

By addressing both functional challenges and consumer preferences, sunflower protein-based films have the potential to become a versatile and sustainable solution in various packaging markets. From eco-friendly food wraps to durable industrial materials, these films can meet diverse needs while contributing to the broader goal of reducing environmental impact in the packaging industry.

In summary, the durability, flexibility, and color of the films are interdependent properties that determine their potential for practical applications. While the addition of cellulose nanoparticles and emulsified oil droplets enhanced certain structural features, such as crack resistance and homogeneity, their impact on mechanical strength and flexibility revealed inherent trade-offs. Films with higher tensile strength and Young’s modulus were less flexible, whereas formulations with greater elongation at break were more susceptible to structural defects like cracks. Similarly, the optical properties, influenced by natural phenolic compounds and emulsion interactions, affect the film’s aesthetic appeal, critical for food packaging. Overall, balancing film strength, flexibility, and color appearance is vital for tailoring their functionality in diverse applications, such as food preservation or biomedical uses, where specific combinations of these properties are prioritized.

Biopolymeric films, unlike standard plastics, are characterized by their ability to absorb moisture and their elevated WVP. To minimize the transfer of moisture between food and its environment, several strategies can be employed [82]. Adding sunflower oil to emulsified protein films has been shown to improve their WVP. The incorporation of oil droplets into the protein matrix forms a lipid network, which reduces the films’ hydrophobicity and consequently their water absorption [83,84,85]. Our findings revealed a great decrease in WVP for E-YSF and E-GSF films compared to those without an oil phase (C-YSF and C-GSF); however, WVP increased with the addition of cellulose nanoparticles. The presence of both cellulose nanoparticles can reduce the hydrophilicity of the protein matrix by forming strong hydrogen bonds between the particles and protein [41]. In this study, however, lipid–protein interactions likely interfered with these bonds, affecting the aggregation of cellulose nanoparticles and enabling greater water permeability through the polymer matrix.

**Figure 7:**
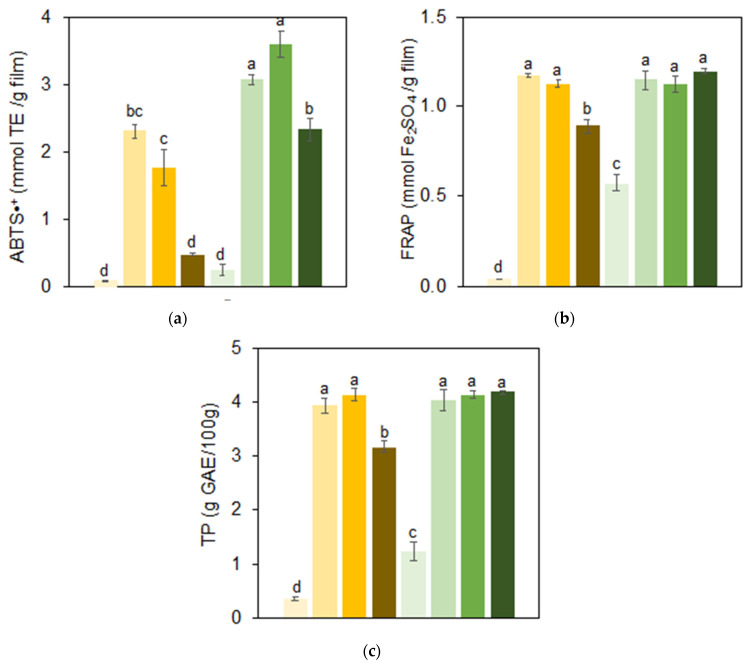
Antioxidant capacity determined by methods (**a**) ABTS•+ and (**b**) FRAP; (**c**) total phenolics (TP) release of films obtained from yellowish (C-YSF, ∎) and greenish (C-GSF, ∎) sunflower protein dispersions, from emulsions prepared only with the yellowish (E-YSF, ∎) or greenish (E-GSF, ∎) sunflower protein dispersions, and from sunflower protein-based emulsions added with the cellulose nanocrystals (E-YSF-CNC, ∎; or E-GSF-CNC, ∎) or cellulose nanofibers (E-YSF-CNF, ∎; or E-GSF-CNF, ∎). Same letters do not show statistical differences (*p* > 0.05).

Figure 7 presents the total phenolic content and antioxidant activity (ABTS•+ and FRAP) of the films. The antioxidant properties were primarily attributed to the natural phenolic compounds in SF, particularly chlorogenic acid, and the presence of eugenol in the emulsified systems (Figure 6). As expected, the C-GSF films exhibited higher phenolic content and antioxidant activity compared to the C-YSF films, due to the higher concentration of phenolic compounds in GSF. The inclusion of eugenol in the emulsion films significantly enhanced antioxidant activity, suggesting that the compound remained stable and was not tightly bound to the polymer matrix or degraded during the drying process. Eugenol, a key phenolic component in clove essential oil, has a molecular structure consisting of two hydrophobic groups (an ester and a propenyl) and an aromatic ring with a hydrophilic hydroxyl group [34]. This structure favors eugenol’s affinity for the oil phase, given its predominantly hydrophobic nature [35]. Films enriched with essential oils often display an amorphous structure with surface cavities, linked to oil evaporation during film formation [2,86]. In our work, the interaction between eugenol, sunflower oil, and the protein interface during the drying process contributed to its retention in the emulsion, thus preserving its antioxidant properties in the final film.

Films containing YSF and CNF particles (E-YSF-CNF) showed a decrease in total phenolic content and antioxidant activity. Despite both cellulose nanofibers and phenolic acids in SF carrying negative zeta potential, they can still interact via non-covalent bonds, hydrogen bonding, and hydrophobic interactions [87,88]. This binding can occur quickly and spontaneously upon contact, within as little as one minute [88,89]. Given the absence of natural phenolic compounds in YSF films, cellulose nanofibers may have bonded to the eugenol molecules, leading to a reduction in the films’ reducing power and radical scavenging activity (Figure 7).

## 4. Conclusions

The results demonstrated that stable emulsions produced with YSF and GSF and cellulose nanoparticles (both fiber and crystal) were well suited for developing films via the casting process. E-GSF, with its high chlorogenic acid content, reduced electrostatic repulsion between protein molecules, leading to larger mean droplet diameters and higher viscosities compared to E-YSF. Emulsion-based films showed significantly lower mechanical properties compared to those made with SF dispersions, likely because of the presence of fissures and tiny voids around the oil droplets. Although E-YSF and E-GSF films had reduced WVP compared to films without an oil phase, the addition of cellulose nanoparticles increased the WVP. The interactions between lipids and proteins in the polymeric matrices negatively impacted the films’ properties, and these effects were not adequately mitigated by the addition of cellulose nanoparticles. Future research should focus on optimizing the film-forming process, including the combined use of cross-linking agents, plasticizers, and nanomaterials to investigate the synergistic effects of different compounds on film structure, with the goal of enhancing mechanical strength and flexibility. Additionally, exploring advanced barrier coatings or integrating hydrophobic additives could reduce WVP and improve moisture resistance. These efforts would expand the functionality and applicability of sunflower protein-based films in diverse packaging markets.

Notably, Eugenol’s affinity for sunflower oil allowed it to be retained in the film-forming emulsion during drying without significant interactions with the polymeric matrix, which notably improved the films’ antioxidant activity. The antioxidant properties of phenolic compounds, including chlorogenic acid and eugenol, make these materials well suited for packaging applications requiring extended shelf life or protection against oxidative degradation, particularly for products like oils, nuts, and dried fruits. Additionally, these packaging can provide tangible health benefits. However, the higher phenolic content in sunflower protein results in darker films, which may limit their appeal in food packaging markets where appearance is critical. Despite this challenge, such films may still find utility in applications where visual appearance is less important, such as bulk or industrial packaging for non-food items. Films based on de-phenolized sunflower protein could be used to develop transparent or lightly tinted films to better meet consumer expectations, especially for premium or visually displayed products.

By balancing functional challenges with consumer preferences, sunflower protein-based films demonstrate significant potential as sustainable packaging solutions for a wide range of markets. From eco-friendly food wraps to durable industrial materials, these films support efforts to reduce environmental impact while expanding their applications in food and other sectors.

## Figures and Tables

**Table 1:** Composition of the film-forming emulsions.

Composition	YSF (5% *w*/*w*)	GSF (5% *w*/*w*)	Glycerol (1.5% *w*/*w*)	Sunflower Oil (3.5% *w*/*w*)	CNC (0.05% *w*/*w*)	CNF (0.05% *w*/*w*)
C-YSF	x		x			
C-GSF		x	x			
E-YSF	x		x	x		
E-GSF		x	x	x		
E-YSF-CNC	x		x	x	x	
E-GSF-CNC		x	x	x	x	
E-YSF-CNF	x		x	x		x
E-GSF-CNF		x	x	x		x

YSF: yellowish sunflower protein; GSF: greenish sunflower protein; CNC: cellulose nanocrystal; and CNF: cellulose nanofiber.

**Table 2:** D_32_, span, zeta potential, and rheological parameters (k: consistency index; n: flow behavior index; η: apparent viscosity) of yellowish (C-YSF) and greenish (C-GSF) sunflower protein dispersions, emulsions prepared only with the yellowish (E-YSF) and greenish (E-GSF) sunflower protein dispersions, and sunflower protein-based emulsions added with cellulose nanocrystals (E-YSF-CNC or E-GSF-CNC) or cellulose nanofibers (E-YSF-CNF or E-GSF-CNF).

Composition	D_32_ (μm)	Span	Zeta Potential (mV)	k (Pa.s^n^)	n	η at 10 s^−1^ (mPa.s)
C-YSF	3.52 ± 0.24 ^a^	1.79 ± 0.05 ^f^	−32.87 ± 0.77 ^a^	0.01 ± <0.01 ^e^	0.87 ± <0.01 ^a^	21.0 ± 0.3 ^e^
E-YSF	0.90 ± 0.01 ^d^	3.39 ± 0.02 ^b^	−35.70 ± 0.26 ^c^	0.03 ± <0.01 ^e^	0.79 ± 0.01 ^b^	29.6 ± 1.8 ^e^
E-YSF-CNC	0.93 ±< 0.01 ^d^	3.61 ± 0.01 ^a^	−36.67 ± 0.67 ^d^	0.05 ± <0.01 ^e^	0.73 ± <0.01 ^c^	35.9 ± 0.6 ^e^
E-YSF-CNF	0.98 ± 0.01 ^d^	3.55 ± 0.04 ^a^	−37.15 ± 0.44 ^d^	0.13 ± <0.01 ^d^	0.67 ± <0.01 ^e^	86.8 ± 8.1 ^d^
C-GSF	2.95 ± 0.05 ^b^	1.86 ± 0.01 ^e^	−33.39 ± 0.95 ^ab^	0.11 ± 0.01 ^d^	0.69 ± <0.01 ^d^	67.5 ± 0.3 ^d^
E-GSF	1.34 ± 0.02 ^c^	2.67 ± 0.02 ^cd^	−33.67 ± 0.71 ^ab^	0.36 ± 0.01 ^c^	0.59 ± <0.01 ^f^	135.2 ± 12.6 ^c^
E-GSF-CNC	1.31 ± 0.02 ^c^	2.73 ± 0.04 ^c^	−34.73 ± 0.88 ^bc^	0.47 ± 0.02 ^b^	0.54 ± <0.01 ^g^	165.6 ± 9.1 ^b^
E-GSF-CNF	1.38 ± 0.01 ^c^	2.63 ± 0.02 ^d^	−34.98 ± 0.97 ^c^	0.68 ± 0.01 ^a^	0.54 ± <0.01 ^g^	296.5 ± 7.6 ^a^

Means with the same letters in the same column do not show statistical differences (*p* > 0.05).

**Table 3:** Thickness, tensile strain, elongation at break, Young’s modulus, and water vapor permeability (WVP) of films obtained from yellowish (C-YSF) and greenish (C-GSF) sunflower protein dispersions, emulsions prepared only with the yellowish (E-YSF) and greenish (E-GSF) sunflower protein dispersions, and sunflower protein-based emulsions added with cellulose nanocrystals (E-YSF-CNC or E-GSF-CNC) or cellulose nanofibers (E-YSF-CNF or E-GSF-CNF).

Film Composition	Thickness (mm)	Tensile Strength (MPa)	Elongation at Break (%) Flexibility	Young’s Modulus (MPa) Rigidez	Water Vapor Permeability (g/m.s.Pa)
C-YSF	0.10 ± <0.01 ^d^	6.0 ± 0.5 ^a^	100.5 ± 8.5 ^b^	0.80 ± 0.05 ^a^	1.15 ± 0.09 ^abc^
E-YSF	0.22 ± 0.02 ^a^	1.5 ± 0.2 ^de^	109.9 ± 5.4 ^bc^	0.14 ± 0.02 ^e^	0.94 ± 0.02 ^c^
E-YSF-CNC	0.16 ± 0.02 ^c^	1.5 ± 0.1 ^e^	92.6 ± 9.0 ^c^	0.15 ± 0.01 ^e^	1.39 ± 0.15 ^ab^
E-YSF-CNF	0.18 ± 0.02 ^b^	1.9 ± <0.1 ^cd^	65.6 ± 9.1 ^d^	0.18 ± 0.02 ^de^	1.47 ± 0.03 ^a^
C-GSF	0.13 ± 0.02 ^d^	4.4 ± 0.3 ^b^	149.7 ± 3.2 ^a^	0.43 ± 0.04 ^b^	1.42 ± 0.18 ^a^
E-GSF	0.19 ± 0.01 ^bc^	2.1 ± <0.1 ^c^	99.1 ± 3.8 ^bc^	0.19 ± 0.02 ^de^	0.99 ± 0.04 ^bc^
E-GSF-CNC	0.21 ± 0.04 ^a^	1.9 ± 0.2 ^c^	62.0 ± 9.4 ^de^	0.22 ± 0.06 ^cd^	1.36 ± 0.05 ^ab^
E-GSF-CNF	0.20 ± 0.01 ^ab^	2.2 ± 0.2 ^c^	52.8 ± 6.4 ^e^	0.25 ± 0.02 ^c^	1.33 ± 0.12 ^abc^

Means with the same letters in the same column do not show statistical differences (*p* > 0.05).

**Table 4:** Color parameters of films obtained from yellowish (C-YSF) and greenish (C-GSF) sunflower protein dispersions, emulsions prepared only with yellowish (E-YSF) and greenish (E-GSF) sunflower protein dispersions, and sunflower protein-based emulsions added with cellulose nanocrystals (E-YSF-CNC or E-GSF-CNC) or cellulose nanofibers (E-YSF-CNF or E-GSF-CNF).

Film Composition	L*	a*	b*	h (°)	C*
C-YSF	10.4 ± 0.7 ^cd^	1.0 ± 0.2 ^d^	0.4 ± <0.1 ^e^	19.5 ± 3.7 ^e^	1.1 ± 0.2 ^e^
E-YSF	26.5 ± 0.9 ^a^	6.9 ± 0.5 ^a^	18.0 ± 0.2 ^a^	69.1 ± 1.4 ^bcd^	19.3 ± 0.1 ^a^
E-YSF-CNC	25.7 ± 0.6 ^a^	5.6 ± 0.9 ^b^	19.2 ± 1.2 ^a^	73.7 ± 2.8 ^abc^	20.0 ± 1.1 ^a^
E-YSF-CNF	26.9 ± 0.6 ^a^	3.6 ± 0.5 ^c^	15.7 ± 0.4 ^b^	77.2 ± 1.8 ^a^	16.1 ± 0.4 ^b^
C-GSF	19.6 ± 0.4 ^b^	2.0 ± 0.1 ^d^	6.3 ± 0.2 ^c^	74.2 ± 4.0 ^ab^	6.6 ± 0.2 ^c^
E-GSF	9.2 ± 0.2 ^d^	0.9 ± 0.1 ^d^	2.1 ± 0.1 ^d^	66.2 ± 3.0 ^cd^	2.3 ± 0.1 ^de^
E-GSF-CNC	11.5 ± 0.6 ^c^	1.2 ± 0.1 ^d^	3.2 ± 0.3 ^d^	69.0 ± 2.5 ^bcd^	3.5 ± 0.3 ^d^
E-GSF-CNF	10.7 ± 0.9 ^cd^	1.0 ± 0.1 ^d^	2.1 ± 0.2 ^d^	65.7 ± 2.2 ^d^	2.3 ± 0.2 ^de^

Means with the same letters in the same column do not show statistical differences (*p* > 0.05).

## Data Availability

The original contributions presented in the study are included in the article/Supplementary Materials, further inquiries can be directed to the corresponding author.

## Supplementary Materials

The following supporting information can be downloaded at: https://www.mdpi.com/article/10.3390/foods14050824/s1, Figure S1: SDS-PAGE electrophoretic patterns under non-reducing (-) or reducing conditions (+, β-mercaptoethanol added) of greenish sunflower protein (G) and yellowish sunflower protein (Y) concentrates; Figure S2: Micrographs of yellowish (C-YSF) and greenish (C-GSF) sunflower protein dispersions and emulsions prepared with yellowish (E-YSF) or greenish (E-GSF) protein dispersions only and added with cellulose nanocrystals (E-YSF-CNC or E-GSF-CNC) or cellulose nanofibers (E-YSF-CNF or E-GSF-CNF).
