# Supplementary material for: Investigating the Impact of Chlorogenic Acid Content and Cellulose Nanoparticles on Sunflower Protein-Based Emulsions and Films
Source: Foods. 2025 Feb 27;14(5):824. doi: 10.3390/foods14050824 (PMC11899123; doi:10.3390/foods14050824)
Supplement: Supplementary file 1 [file foods-14-00824-s001.zip › foods-3405731-supplementary.pdf]

Investigating the Impact of Chlorogenic Acid Content and Cellulose Nanoparticles on Sunflower Protein-Based Emulsions and Films

Andresa Gomes [1,2,*], Lais Brito Cangussu [3], Rosiane Lopes Cunha [4], Leandro Soares de Oliveira [5,6], Adriana Silva Franca [5,6] and Ana Letícia Rodrigues Costa [7]

[1] Department of Food Engineering, School of Animal Science and Food Engineering, University of São Paulo (USP), Av. Duque de Caxias Norte, 225, Pirassununga 13635-900, SP, Brazil
[2] Food Research Center (FoRC), University of São Paulo, Rua do Lago, 250, Semi-Industrial Building, Block C; São Paulo 05508-080, SP, Brazil
[3] Instituto Federal do Mato Grosso do Sul (IFMS), Rua Salime Tanure, S/N, Coxim 79400-000, MS, Brazil; lais.cangussu@ifms.edu.br (L.B.C.)
[4] Department of Food Engineering and Technology, School of Food Engineering, University of Campinas (UNICAMP), Rua Monteiro Lobato, 80, Campinas 13083-862, SP, Brazil; rosiane@unicamp.br
[5] Programa de Pós-Graduação em Ciência de Alimentos (PPGCA), Universidade Federal de Minas Gerais, Av. Antônio Carlos, 6627, Belo Horizonte 31270-901, MG, Brazil; leandro@demec.ufmg.br (L.S.O.); adriana@demec.ufmg.br (A.S.F.)
[6] Departamento de Engenharia Mecânica (DEMEC), Universidade Federal de Minas Gerais (UFMG), Av. Antônio Carlos, 6627, Belo Horizonte 31270-901, MG, Brazil
[7] Institute of Exact and Technological Sciences, Campus Florestal, Federal University of Vicosa (UFV), Rodovia LMG 818, km 6, Florestal 35690-000, MG, Brazil; ana.leticia@ufv.br
* Correspondence: andresa.gomes@usp.br or andresagomeseng@gmail.com

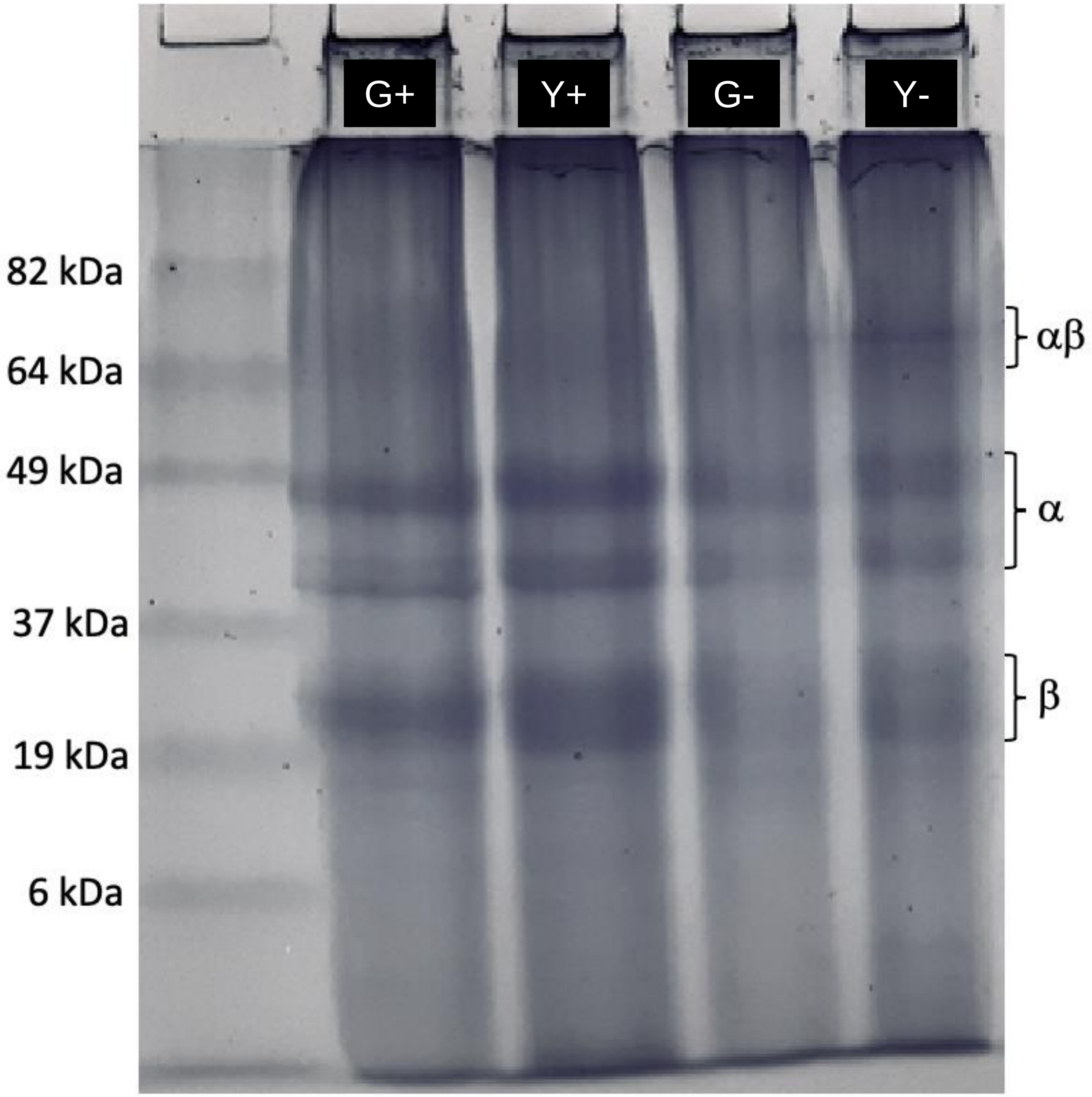

Figure S1. SDS-PAGE electrophoretic patterns under non-reducing (-) or reducing conditions (+, -mercaptoethanol added) of greenish sunflower protein (G) and yellowish sunflower protein (Y) concentrates.

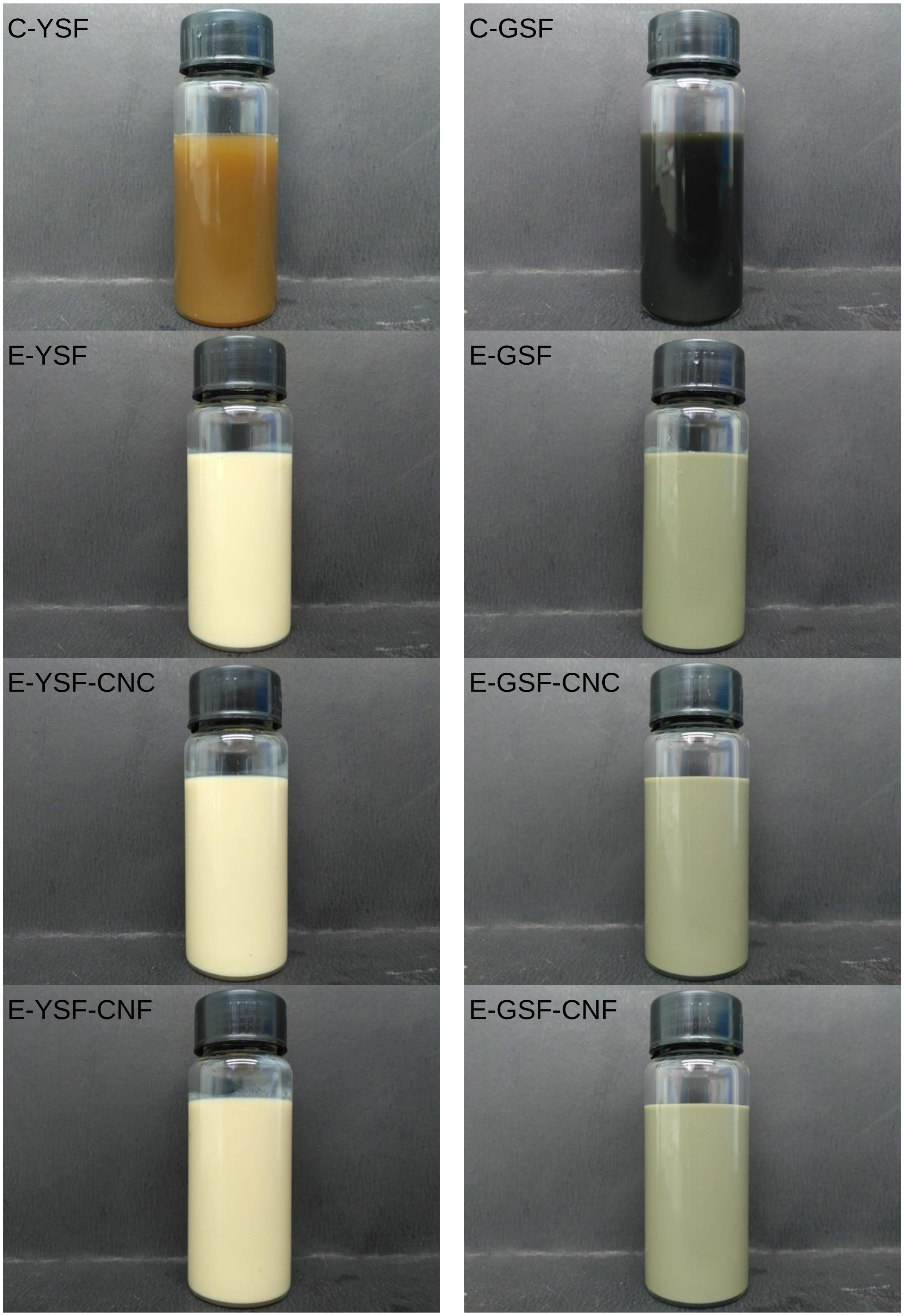

Figure S2. Micrographs of yellowish (C-YSF) and greenish (C-GSF) sunflower protein dispersions and emulsions prepared with yellowish (E-YSF) or greenish (E-GSF) protein dispersions only and added with cellulose nanocrystals (E-YSF-CNC or E-GSF-CNC) or cellulose nanofibers (E-YSF-CNF or E-GSF-CNF).
